# The Tri-State Medical Convention of Alabama, Georgia and Tennessee

**Published:** 1890-11

**Authors:** D. H. Howell, W. L. Gahagan


### THE TRI-STATE MEDICAL CONVENTION OF ALABAMA, GEORGIA AND TENNESSEE.

The Convention was opened October 14, in Turner Hall, by prayer by the Rev. Charles Hyde. President J. B. Cowan in the chair.

A committee of five was appointed to revise the constitution and by-laws.

The secretary reported a membership of 87 at the opening of this session.

The treasurer's report was then read and referred to the auditing committee.

Committee on credentials reported 30 names, and they were elected to membership.

At the afternoon session the following papers were read:

A case of Cancrum Oris, or Gangrenous Stomatitis, by W. P. McDonald, M. D.

Dr. E. E. Kerr, a case of Inflamed Inguinal Glands. Discussed by Drs. Townes, Smith and Gahagan.

Dr. James E. Reeves: On All Sides a Learned Doctor. Discussed by Drs. Parks, Drake, Boyd, Baxter and Prince.

Dr. Andrew Boyd: Report of a case of Fracture of the Elbow Joint. Discussed by Dr. Baxter.

Mr. Sydney B. Wright: A paper on Expert Testimony. Discussed by Drs. Smith, Camp, Baxter, Cobleigh and Cowan.

Dr. W. O. Bogard gave a report of a case of Neurosis in which there were attacks resembling Epilepsy.

Dr. W. L. Gahagan: Neuralgia. Read by title.

October 15.—Called to order by the president. Opening prayer by the Rev. Dr. J. W. Willingham.

Minutes of the first day's session were then read by Secretary *pro tem.* Gahagan, and adopted.

Telegram from Dr. J. B. S. Holmes, also letter from Dr. J. E. Purden, regretting their absence.

The reading of papers was then resumed.

Dr. J. C. Shapard: A Few Remarks on the Fevers of Middle Tennessee, and their Treatment.

Dr. Llewellyn P. Barber read a Contribution to the Study of the Continued Fevers of the South. These two papers were discussed together, by Drs. Sims, Park, Drake, Reeves, Long, Murfree, Baxter, Cowan, Douglass and Berlin.

Dr. G. A. Baxter presented a paper on Some New Uses of Silicate of Soda in Surgery. This paper called forth a good deal of discussion from Drs. Murfree, Douglass, Howell and Wells.

Dr. Richard Douglass read a paper on Abscess of the Liver.

Dr. E. E. Kerr read a paper entitled Report of a Case of Gall Stones. Both these papers were discussed by Drs. Drake' Crumley and Berlin.

The election of officers came next in order, and the following were elected:

President—Robert Battey, Rome, Ga.

First Vice-President—E. T. Camp, Gadsden, Ala.

Second Vice-President—Richard Douglass, Nashville, Tenn.

Third Vice-President—Dan. H. Howell, Atlanta, Ga.

Secretary—Frank Trester Smith, Chattanooga, Tenn.

Treasurer—R. E. West, Chattanooga, Tenn.

Dr. J. B. Murfree presented a paper on Uterine Fibroma. Discussed by Drs. Wathen and Douglass.

Dr. W. H. Wathen read a very able paper on Laparotomy in Ectopic Pregnancy.

On motion of Dr. Douglass, a vote of thanks was tendered Dr. Wathen for his valuable paper.

The session then adjourned to meet at the Stone Church at 7:30 o'clock p. m.

At the Stone Church an address of welcome was delivered by Dr. G. W. Drake, and responded to by Dr. G. C. Savage.

Following this, the president delivered his address, the subject being "The Doctor."

Later in the evening Dr. and Mrs. Baxter tendered the Association a reception at their residence.

October 16.—President called meeting to order at 10 a. m. The minutes of the second day were read and approved.

The president appointed Drs. Harbin, Camp and Gahagan a committee to prepare resolutions on the death of Dr. V. J. Kennedy, of Sale Creek, Tennessee.

The committee on revision reported a new constitution. This was read and laid over for one year.

Dr. T. H. Wood presented a paper on Hypertrophy of the Tonsils. Discussed by Drs. Gahagan, Smith, Savage, Westmoreland Reeves and Steele.

A case of Remarkable Injury, with Recovery, was read by Dr. E. A. Cobleigh; discussed by Drs. Drake, Reeves, Wilson and Camp.

Dr. W. F. Westmoreland read a paper on Morbid Reflex Neurosis Amenable to Surgical Treatment; discussee by Drs. Howell, Perkins, Stewart, Cowan and Reeves.

Dr. Crumley presented a case Resembling Epilepsy, to be examined by the members.

Dr. G. R. Rathmell reported a case of Typhoid Fever, Dr. W. C. Townes showed the specimens from the post mortem on the above case, and also read a paper on Dilated Cardiac Hypertrophy with Nephritic Complications, with the enlarged heart and kidney from a case.

Dr. Howell proposed Atlanta as the next place of meeting, but the motion was lost.

The following committees were appointed:

On Arrangements—Smith, Bogart, Gahagan.

Credentials—Stewart, Mills, Boyd.

Auditing—Wise, Hunt, Russey.

At the evening session Dr. R. J. Tripp read the report of a case of Peritonitis.

Dr. C. H. Holland read a report of a case of Phlegmonous Abscess; discussed by Drs. Westmoreland, Baxter and Stewart.

Mr. Eli S. Reed asked the Association if the running of the sewing machine by the treadle was injurious to the health of women. This was discussed pro and con by Drs. Wells, Drake, Bogart, Baxter, Smith and Crumley.

The following papers were read by title: Report of a case of Ovariotomy, by Dr. J. H. Atlee; Fluorescin in the Diagnosis of Corneal Affections, by Dr. Frank Trester Smith; the Dynamics of Mediumism, by Dr. J. E. Purdon.

Report of a case of Abscess of the Liver, by Dr. J. R. Rathmell.

Stricture of the Urethra and its Complications, by Dr. J. D. Gibson.

Notes on Apostoli's Method of Treating Uterine Fibroma, by Plym S. Hayes.

The resolutions on the death of Dr. V. J. Kennedy were adopted.

The following was also adopted:

Resolved, That we hereby tender a vote of thanks to our retiring president for the able and just manner in which he has presided over the Association, and for his very able address; to the hotels and railroads for reduced rates; to Dr. and Mrs. Baxter for their pleasant reception; to the exhibitors for their presence and courtesies; to the members, and the Chattanooga press for their courtesies and kindly notices, and to the trustees of the Stone Church for the use of the auditorium for the president's address.

D. H. HOWELL.
W. L. GAHAGAN.

The president thanked the Association for the manner in which it had supported him; also, the McIntosh Battery Co., for the gavel, and declared the Association adjourned to meet in Chattanooga next fall.

Over sixty members were added to the membership at this meeting.

